# Depth-dependent microskeletal features modify light harvesting in *Turbinaria reniformis* corals

**DOI:** 10.1016/j.isci.2025.113137

**Published:** 2025-07-17

**Authors:** Netanel Kramer, Claudia Tatiana Galindo-Martínez, Steven L. Jacques, Martin Tresguerres, Yossi Loya, Daniel Wangpraseurt

**Affiliations:** 1 Marine Biology Research Division, Scripps Institution of Oceanography, University of California, San Diego, San Diego, CA, USA; 2 Department of Bioengineering, University of Washington, Seattle, WA, USA; 3 School of Zoology, Tel-Aviv University, Tel Aviv, Israel

**Keywords:** Aquatic biology, Biological sciences, Marine organism, Zoology

## Abstract

Coral skeletal morphology modulates light exposure in symbiotic algae, especially in light-limited environments like mesophotic reefs. However, quantifying light capture within complex coral structures remains challenging. Here, we used optical coherence tomography and high-resolution X-ray scanning to explore depth-dependent bio-optical properties of shallow and mesophotic *Turbinaria reniformis* corals from the Gulf of Eilat/Aqaba, Red Sea. We identified two distinct skeletal layers: a highly scattering superficial layer and a deeper, more light-penetrating layer. Mesophotic corals showed higher scattering coefficients and a lower anisotropy of scattering values, yielding increased reflectivity. Regardless of depth, coenosteum grooves facilitated forward scattering, while protruding features such as spines and septa increased surface reflectivity and isotropic scattering. Light simulations demonstrated an enhanced fluence rate at the skeleton-water interface, with mesophotic corals enhancing the available light up to 2.7-fold. These findings suggest that microskeletal heterogeneity fine-tunes light capture at the microenvironmental scale, thereby enhancing light-harvesting efficiency across depth.

## Highlights

• Coral skeletons include a top scattering surface and a light-penetrating layer
• Mesophotic coral skeletons enhance light capture through increased reflectivity
• Microscale skeletal features modulate local light distribution within corals

## Graphical abstract

**Figure:**
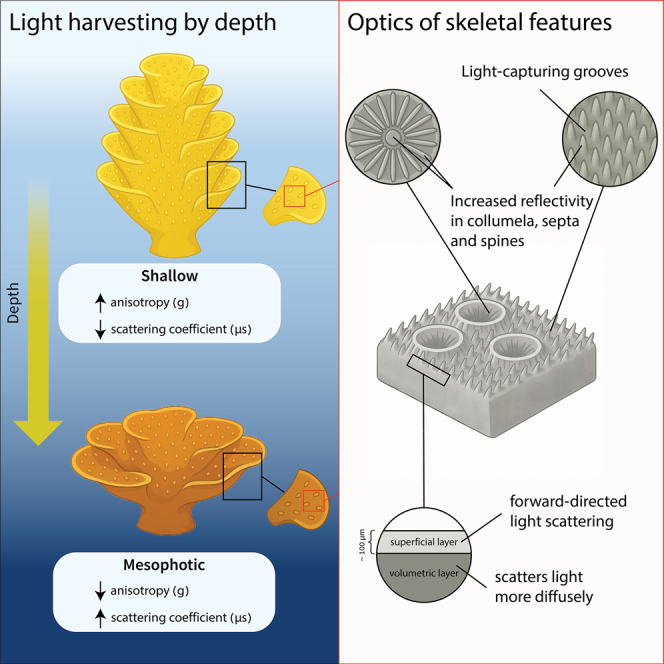

## Introduction

Corals serve as the foundational species that construct the three-dimensional (3D) framework of tropical reef ecosystems.^1^ Their structural diversity varies widely across natural environmental gradients, shaping reef habitats and influencing ecological processes.^2^^,^^3^ Coral skeletal architecture is shaped by multiple interacting factors, including light availability, nutrient levels, hydrodynamic conditions, and spatial competition.^4^^,^^5^^,^^6^^,^^7^ In shallow-water reefs, corals exhibit complex self-shading morphologies to photoadapt to the prevailing high-light conditions.^4^^,^^8^^,^^9^^,^^10^^,^^11^ In contrast, corals in deeper waters, such as the mesophotic zone (typically below 30 m), often adopt flatter colony structures to enhance light capture in low-light environments.^7^^,^^12^

Beyond depth-related adaptations, the three-dimensional nature of coral skeletons creates localized microenvironmental gradients within a single colony, influencing the morphology and physiology of individual polyps despite their shared genetic identity.^13^^,^^14^^,^^15^ For example, variations in light exposure within branching colonies can trigger adaptive changes in individual polyps, leading to spatial heterogeneity in photosynthetic performance and microskeletal morphology.^14^

While coral architectural adaptations are well documented, the role of their fine-scale skeletal features in light-harvesting remains unclear. The coral skeleton is composed of distinct structural features, including corallites (the skeletal cups housing individual polyps), the coenosteum (the skeletal area between corallites), and fine-scale features, such as spines and grooves, which contribute to microstructural complexity. However, studying their light-harvesting function is challenging due to the lack of tools capable of accurately quantifying the complexity of coral structures across different scales. Earlier studies employed simplified 2D or 3D optical models to explore how morphology influences light interception.^4^^,^^9^^,^^16^^,^^17^^,^^18^^,^^19^^,^^20^ However, these models relied on schematic representations that failed to capture the intricate details of natural coral architectures. Therefore, a precise understanding of how these complex structures enhance light capture and contribute to coral growth remains a challenge, highlighting the need for refined methodologies in coral photobiology research.

Recent advancements in digital imaging and computational modeling have significantly expanded the scope of 3D-based coral reef studies.^8^^,^^21^^,^^22^^,^^23^ While photogrammetry and 3D laser scanning provide cost-effective, large-scale structural data, they are limited to external surface imaging and lack the spatial resolution to capture fine skeletal details. In contrast, micro-computed tomography (μCT) offers high-resolution imaging of both internal and external skeletal elements, enabling precise quantification of skeletal features.^8^^,^^23^^,^^24^ The latest breakthrough in high-resolution coral imaging is optical coherence tomography (OCT), a non-invasive technique using near-infrared radiation (NIR) to visualize live coral structures in detail.^25^^,^^26^^,^^27^^,^^28^ Furthermore, recent advances employ voxel-based Monte Carlo photon simulations on high-resolution coral meshes,^8^^,^^19^^,^^29^^,^^30^^,^^31^ assigning each voxel its local inherent optical property values. Such voxel-based or mesh-based approaches are uniquely capable of mapping light fields over the surface and inside microscale skeletal compartments—regions that are effectively inaccessible to direct experimental measurement.

This study investigates how *Turbinaria reniformis* corals modulate light exposure across shallow and mesophotic environments, with a focus on their microscale skeletal structures. We employed OCT and μCT to obtain high-resolution 3D structural and optical data from *T. reniformis* specimens collected from the Gulf of Eilat/Aqaba, Red Sea. Additionally, we applied voxel-based Monte Carlo light simulations to model light transport within coral structures. These findings reveal that depth-dependent variations in microskeletal architecture and optical properties play a vital role in modulating internal light fields on and within coral skeletons. This modulation likely enhances light availability for symbiotic algae, particularly under low-light mesophotic conditions, thereby supporting depth-specific optimization of photosynthesis and skeletal growth.

## Results

### Optical reflectance, scattering, and anisotropy

**Figure 1:**
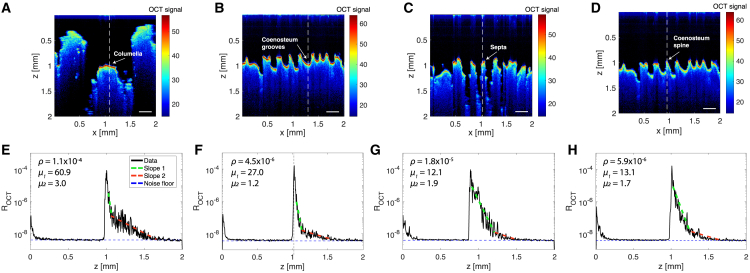
Optical coherence tomography (OCT) imaging and reflectance profiles of coral microstructures (A–D) Cross-sectional OCT images of *T. reniformis*, highlighting distinct structural features: (A) columella, (B) coenosteum grooves, (C), septa and (D) coenosteum spines. The color scale indicates OCT signal intensity (dB). Scale bars, 250 μm. The vertical white dashed lines indicate the regions corresponding to the reflectance profiles shown below. (E–H) Corresponding corrected reflectance (*R*_*OCT*_; A-scan) profiles along the *z* axis for each coral structure. The key optical parameters, including the total attenuation coefficient (*μ*_1_ and *μ*_2_) corresponding to the slopes for the two skeletal layers (superficial and volumetric), and local reflectivity (*ρ*), were noted for each profile.

**Figure 2:**
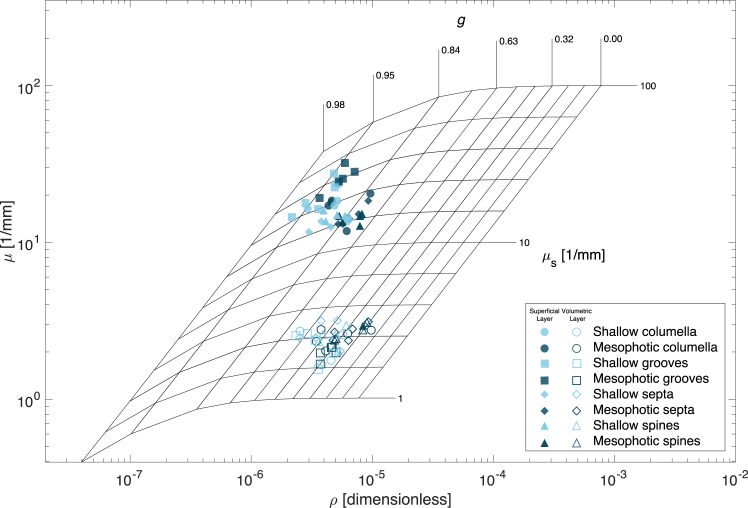
Extracted optical properties (*μ* and *ρ*) from OCT signals of coral skeletal components mapped onto a lookup *μ*_*s*_-*g* grid Markers represent the median per sample for different skeletal features from shallow (*light blue*) and mesophotic (*dark blue*) depths, with filled symbols indicating superficial skeletal layer properties and open symbols indicating volumetric skeletal layer properties.

**Figure 3:**
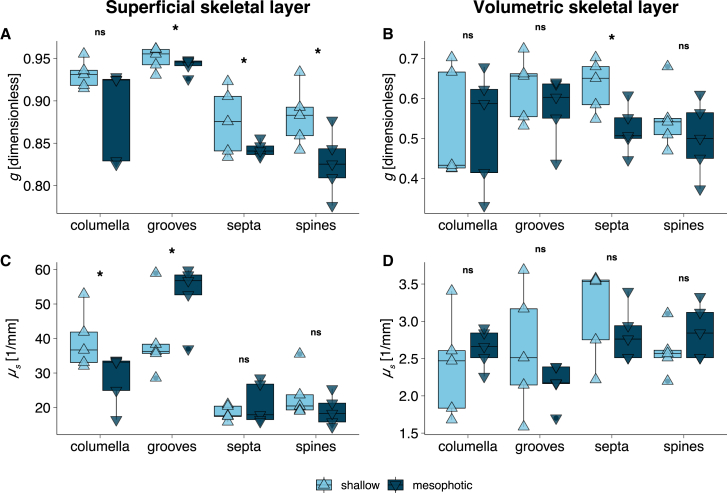
Scattering properties of morphological skeletal components between shallow (*light blue*; triangle point up) and mesophotic (*dark blue*; triangle point down) *T. reniformis* for (A + C) the superficial skeletal layer and (B + D) the volumetric skeletal layer (A and B) The anisotropy of scattering (*g*) across four skeletal features: columella, grooves, septa, and spines. (C and D) Scattering coefficient (*μ*_*s*_; 1/mm) measurements for the same skeletal features. Each triangle represents a sample median. Boxplots horizontal lines depict the median, box height depicts the interquartile range, and whiskers depict ±1.5× interquartile range. Asterisk denotes significance (*Hg*, CI_95%_; 5,000 bootstrap resamples).

The optical analysis of *T. reniformis* from OCT scans revealed significant depth-dependent variations in the scattering coefficient (*μ*_*s*_), and anisotropy of scattering value (*g*) across different skeletal features (MEPA, *p* < 0.001; Figures 1, 2, and 3; Table 1). We identified clear transition points where the slope of the OCT signal (i.e., *μ*) changed, corresponding to the boundary between a superficial, highly scattering surface layer and a deeper, more light-transmissive skeletal layer (Figures S4 and S5). Within the first ∼100 μm of the skeleton, the OCT signal showed high initial reflectance followed by rapid attenuation, indicating strong backscattering near the surface. Deeper into the skeleton, attenuation decreased, suggesting a transition to more forward-scattering and less optically dense structures, until the signal reached the noise floor (Figures 1 and S4). The light reaching the second layer of the skeleton was partially attenuated as it passed through the first layer (∼100 μm thick). This attenuation was due to both the specular reflectance at the air-skeleton interface and the exponential decay of light intensity within the superficial 100 μm layer. The amount of light entering the deeper skeletal layer was therefore reduced by these combined effects, and this was taken into account when analyzing the optical properties of the bulk skeleton. The transition point (Figure S4A) marks the boundary between the highly scattering superficial layer and the more light-penetrating volumetric skeletal layer, as indicated by the lower *g* values. Consequently, the optical properties for each skeletal layer were extracted (Figure 2).

**Table 1:** Optical parameter table

Parameter	Symbol	Unit	Definition
Local reflectivity	*ρ*	Dimensionless	Ratio of reflected to incident light intensity
Total attenuation coefficient	*μ*	cm^−1^	Rate of intensity decrease due to absorption and scattering
Absorption coefficient	*μ*_*a*_	cm^−1^	Probability of absorption per unit length
Scattering coefficient	*μ*_*s*_	cm^−1^	Probability of scattering per unit length
Anisotropy factor	*g*	Dimensionless	Average cosine of the scattering angle
Diffuse spectral reflectance	*R*_*d*_	Dimensionless	Fraction of incident light diffusely reflected, considering scattering in all directions

For both skeletal layers in all skeletal features, mesophotic *T. reniformis* consistently exhibited high *ρ*, corresponding to lower *g* values compared to their shallow counterparts (Figures 2; 3A, 3C, and S5A). However, specific depth-dependent differences among the skeletal features varied for both layers. *g* exhibited a consistent hierarchical pattern across both depths, with spines demonstrating the lowest *g*, followed by the septa, columella, and grooves. For example, in the superficial skeletal layer, spines exhibited a significant 5% increase in *g* from mesophotic to shallow **(***g* **=** 0.83 vs. 0.87; *Hg* = −1.38 [CI_95%_: −0.266; −0.10]; Figure 3A). Among the skeletal features, the coenosteum grooves showed the highest *g* values for both depths, reaching an anisotropy factor of 0.96 in the superficial skeletal layer and 0.7 in the volumetric skeletal layer.

The *μ*_*s*_ was found to be an order of magnitude higher in the superficial layers compared to the volumetric layer in corals from all depths. Between depths, no significant differences were detected in the spines and the septa features. In contrast, the columella and grooves exhibited pronounced differences between depths. The superficial layer of the grooves had a 34% higher *μ*_*s*_ in mesophotic corals compared to their shallow counterparts **(***μ*_*s*_ **=** 52.92 ± 3.20 vs. 39.56 ± 2.63 mm^−1^; median ±SE; *Hg* = 1.23 [CI_95%_: 0.34; 2.07]; Figure 3C). Additionally, *μ*_*s*_ values in the superficial layer of the grooves were more than double those of the other elements. This pattern was reversed for the volumetric skeletal layer, where the coenosteum spines and septa showed higher scattering relative to the columella and grooves, with *μ*_*s*_ ranging from 2.57–3.54 mm^−1^ (Figure 2D). However, the difference between shallow and mesophotic corals was less pronounced and not significant (MEPA, *p* = 0.62).

**Figure 4:**
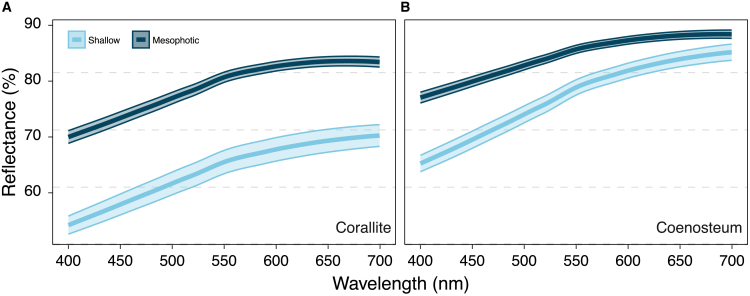
Diffuse spectral irradiance (%) at 400–700 nm over the skeleton surface of *T. reniformis* Measurements were taken over (A) Corallites and (B) Coenosteum between shallow (*light blue*) and mesophotic (*dark blue*) colonies. The shaded areas represent the standard errors.

The differences in diffuse reflectance (*R*_*d*_; Figure 4) aligned with the observed patterns in *ρ* (Figure S5A), with skeletons from mesophotic corals exhibiting significantly higher and less variable *R*_*d*_ than skeletons from shallow corals (MEPA, *p* < 0.01). In the coenosteum, mesophotic samples exhibited 4% higher reflectance compared to shallow samples, whereas mesophotic corallites reflected 14% more light than their shallow counterparts. Additionally, in skeletons from both depths, *R*_*d*_ from the coenosteum was higher than that over the corallites (*Hg*_*shallow*_ = 2.17 [CI_95%_ 1.87; 2.47]; *Hg*_*mesophotic*_ = 1.62 [CI_95%_: 1.29; 1.95]), with values as low as 56.3% in shallow corallites (Figure 4A) and reaching up to 92.2% in the mesophotic coenosteum (Figure 4B).

**Figure 5:**
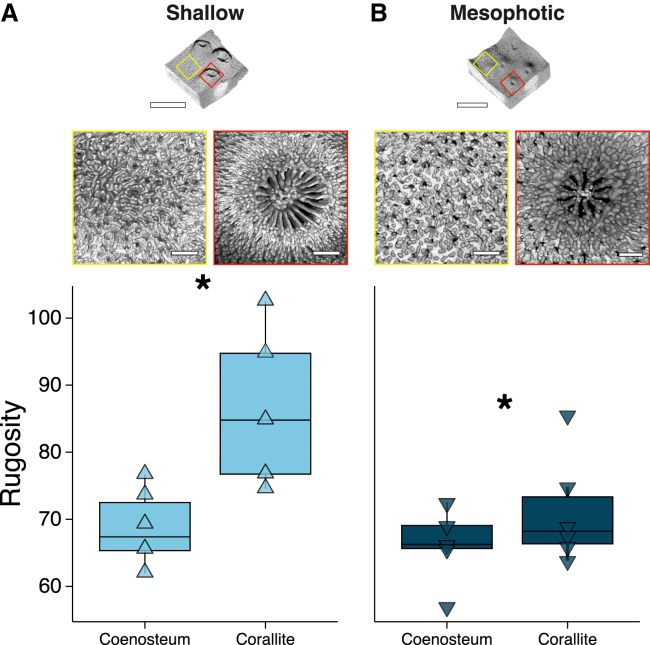
Skeletal rugosity in *T. reniformis* corals (A) shallow (*light blue*; triangle point up) and (B) mesophotic (*dark blue*; triangle point down) measurements in the coenosteum and corallite regions. The top panels display the 3D surface renderings of the coral skeletons (scale bars, 5 mm) with corresponding μCT images (scale bars, 0.5 mm) highlighting the coenosteum (yellow box) and corallite (red box) regions. Bottom panels display boxplots of rugosity for each depth and region. Each triangle represents a sample median. Horizontal lines depict the median, box height depicts the interquartile range, whiskers depict ±1.5× interquartile range. Asterisk denotes significance (*Hg*, CI_95%_; 5,000 bootstrap resamples).

Lastly, the rugosity index values (Figure 5) revealed corallites from shallow corals had a 23% significantly higher structural complexity compared to those from mesophotic corals (*Hg* = 1.41 [CI_95%_: 0.77; 1.95]); however, no significant differences were observed in the coenosteum between the depths (*Hg* = −0.02 [CI_95%_: −1.10; 1.17]).

### 3D Monte Carlo simulations

**Figure 6:**
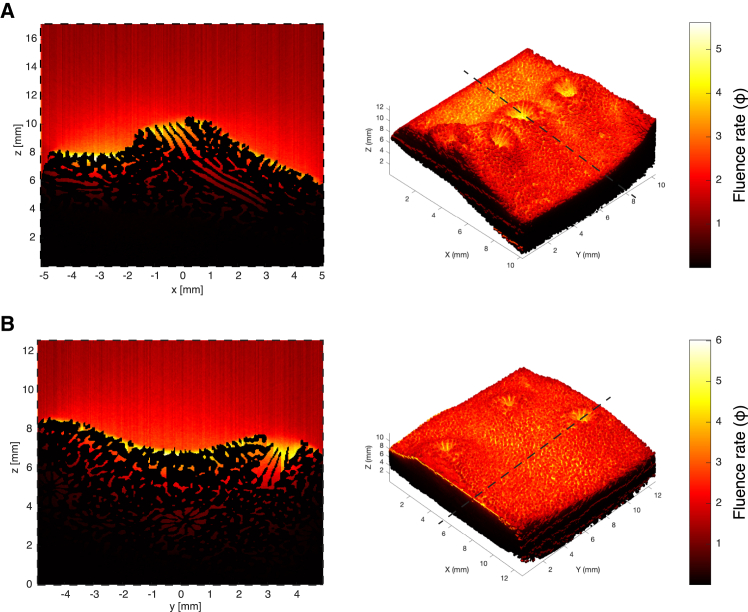
Fluence rate (ϕ) distributions based on Monte Carlo simulations (A) shallow and (B) mesophotic *T. reniformis* morphotypes. The right panels display 3D skeletal surface mapping of fluence rate with dashed lines indicating the position of vertical cross-sections shown in the left panels, which illustrate the light field distribution in the surrounding water layer. The fluence rate values were normalized to the incident light (ϕ = 1).

Using the extracted optical values from the OCT, we ran Monte Carlo light simulations that further revealed substantial differences in the fluence rate (Φ; Figure 6; Table S1) between morphotypes. We generated fluence rate distributions for each layer (water, superficial coral skeleton, and volumetric coral skeleton). The data were transformed to a logarithmic scale to normalize the distributions and increase the resolution within smaller fluence rate values. Overall, the fluence rates were consistently higher in the superficial skeletal layers than in the volumetric skeletal layer, with mesophotic samples generally exhibiting greater fluence rates and sharper distribution peaks across all layers (Figure 6). Furthermore, the mesophotic deeper skeletal layer exhibited reduced skewness and kurtosis, indicating a more symmetrical fluence distribution within the skeleton than its shallow counterparts (Table S1). All simulations demonstrated an enhanced fluence rate over the coenosteum groove surfaces and calyx areas, with the highest values observed at the skeleton-water interface, reaching up to 2.7 times the incident light in the mesophotic models, as indicated by the 95th percentile (p95) values.

## Discussion

Understanding the mechanisms that allow coral to adapt to different light environments is essential for understanding their survival, growth, and reproduction strategies and how they might be impacted by environmental change.^32^^,^^33^ The optical properties of coral skeletons play a crucial role in optimizing light distribution for photosynthesis, particularly in mesophotic environments where light availability is limited. These properties regulate light capture and enhance the symbiotic relationship between corals and their endosymbiotic microalgae, which rely on photosynthetically active radiation. Our results reveal distinct skeletal and optical modifications of *Turbinaria reniformis* to the contrasting conditions typical of shallow and mesophotic environments, particularly at the microscopic scale.

In most of the OCT scans, we noticed a distinct shift in the optical properties between the superficial and volumetric skeletal layers, indicating different optical properties in these regions (Figure 1). The superficial layer (∼100 μm thick) consistently exhibited strong lateral scattering and light redirection (Figures S5A and S5C). Our data confirm that while the superficial layer modulates light interactions at the initial boundary, the volumetric skeleton is the dominant contributor to overall light reflectance. This is due to a higher reduced scattering coefficient of the deeper parts of the skeleton [*μ*_*s*_(1−*g*)], which governs the total diffuse reflectance from the skeleton.^34^ The superficial layer, which first interacts with incoming light (after the tissue layer), is distinct from the volumetric skeleton probably due to differences in skeletal density, crystal structure, and/or compositional architecture.^8^^,^^20^^,^^35^^,^^36^^,^^37^ These differences are largely governed by coral biomineralization processes: skeletal formation begins with amorphous calcium carbonate precursors produced inside the calcifying cells, which are secreted into the tissue-skeleton interface and attach to the growing skeleton, where they remain amorphous for several hours before crystallizing into aragonite.^38^ During this process, the deposition of protein-bound aragonite crystals often forms spherical or spindle-shaped structures that shape the skeletal architecture. These nano-to microscale features likely contribute to the observed variability in optical properties, ultimately influencing how photons are directed toward shaded symbiotic algae within the tissue.^39^ In contrast, deeper skeletal layers exhibited lower *μ*_*s*_ and lower *g* values, indicating more diffuse scattering (Figures 2; 3B, and 3D), generally behaving similar to the scenario shown in Figure S6D compared to the superficial layer. These properties enable light to penetrate deeper into the skeleton, which can sustain photosynthesis of symbiotic dinoflagellates in tissue layers further from the surface, particularly in imperforate skeletons, or potentially support photosynthetic endolithic communities.^37^

Compared to mesophotic samples, shallow corals exhibited increased inter-individual variation in skeletal bulk reflectivity (*R*_*d*_), with standard deviations more than double in corallite regions (SD = 8.09 vs. 3.60; Figure 4). This pattern could potentially be due to frequent environmental fluctuations in shallow light regimes^32^ that may affect photosynthesis with rippling effects on coral metabolism, tissue growth, and biomineralization that affect skeletal properties. In contrast, the skeleton of mesophotic corals exhibited higher and less variable reflectivity, likely due to more stable environmental conditions at depth (e.g., light, temperature, and nutrient levels), and maintained more consistent isotropic scattering across colonies (Figures 3A and 4). Such characteristics optimize light distribution under dim conditions, ensuring sufficient light harvesting by the photosymbionts in mesophotic environments.^9^^,^^40^ The OCT analysis confirmed that increased isotropic scattering in mesophotic skeletons resulted in a broader surface light distribution, although this modification leads to rapid light attenuation and reduced penetration depth. Furthermore, these optical properties match the thinner skeletal geometries often found in mesophotic corals,^7^^,^^8^^,^^35^ representing a coordinated adaptation that maximizes light utilization in low-light environments.

At the microscale, *T. reniformis* exhibits a “papillose-like” coenosteum surface texture characterized by numerous spines which form convex spaces (i.e., grooves), regardless of depth (Figure 5). Features such as coenosteum grooves showed the highest *μ*_*s*_ and *g* values (Figures 2 and 3), indicating forward-directed scattering that facilitates deeper light propagation through the skeleton. These grooves likely function as light traps, capturing and channeling light to specific areas that would otherwise be exposed to suboptimal conditions, thereby enhancing localized photosynthesis (Figures 3 and 6). Conversely, protruding structures, such as spines and columella, showed lower *μ*_*s*_ and *g* values, resulting in more isotropic scattering and higher reflectivity. These traits compensate for a smaller surface area by increasing reflectivity and lateral scattering (Figure S5).

At the macroscale, enhanced light capture efficiency and potential productivity are often linked to coral morphological adaptations, including branch flattening^41^ and shifts from mounding to plating forms.^42^ These modifications are readily evident in mesophotic zones, where plating morphologies prevail.^12^^,^^43^ Like many depth-generalist corals, *T. reniformis* exhibits variations in its overall morphology when exposed to their light environments.^44^ In shallow waters, colonies display vertically oriented, narrowly spaced plates, a morphology that protects their algal endosymbionts from excessive light and heat. In contrast, corals inhabiting low-light demonstrate a flattened morphology with more widely spaced plates, which is an energetically efficient adaptation for maximizing light capture in low-light environments.^4^^,^^45^ Corals with plocoid corallite morphology, such as our studied *T. reniformis*, in which each polyp possesses its own skeletal wall, characteristically develop a more coenosteum-dominated structure in low-light environments with associated smaller, more widely spaced corallites and an increased coenosteum area.^8^^,^^17^^,^^46^ Matching those observations, our rugosity analysis revealed a significantly greater microscale surface complexity in shallow corallites, which had up to 2-fold higher rugosity than their mesophotic counterparts (Figure 5). This pattern is typical in shallow-water skeletons, as it creates more self-shading surfaces to protect photosymbionts from photodamage.^15^^,^^42^^,^^47^ Thus, the lower rugosity in the coenosteum-dominated mesophotic corals facilitates efficient light capture and minimizes scattering losses in their dim environment. Furthermore, consistent with previous investigations^9^^,^^20^ and our current optical results, the lower rugosity observed in coenosteum regions suggests these structures function to optimize light reflection and channel light more effectively into the polyps.

Monte Carlo simulations of light propagation provided further insights into how skeletal optical properties influence light capture at different depths (Figure 6). By visualizing areas of high and low light intensity, and shadowing effects, we reveal how the interplay between coral microstructure and optical traits creates a microscale light environment that enhances photosynthesis. To further explore these properties, we conducted simulations on a flat surface mimicking varying *μ*_*s*_ and *g* scenarios, which confirmed their impact on light propagation and fluence rates (Figure S6). These findings highlight the importance of skeletal architecture on modulating light availability, complementing the known role of coral tissue in shaping internal light fields.^48^

Relative to the coral tissue, which is a strongly light-scattering matrix, the skeleton has a lower scattering coefficient which allows efficient light propagation to the shaded tissues.^19^ Correspondingly, we noticed light enhancement at the skeleton-water interface, with pronounced differences between depths, whereby the skeleton from mesophotic corals backscatter more light back into the water layer compared to shallow corals (Figure 6; Table S1). Moreover, by using Monte Carlo simulations, we gained insights into how light propagates within the coral skeleton in a non-invasive manner. Generally, at both depths we observed a heterogeneous light field over the skeleton surface, with a more homogeneous distribution of light within the skeleton pores (Figure 6). However, mesophotic corals exhibited a relatively more uniform distribution of light compared to shallow samples, likely reflecting adaptations of mesophotic corals to optimize light harvesting under low-light conditions by avoiding extreme fluence rate variability.

Clearly, there are other complex interdependencies between irradiance, reef corals, and photosynthesis, which are beyond the scope of this study and require further investigation. Although our study primarily focused on skeletal optical properties, the role of the various coral tissue layers is crucial for determining how light is absorbed and utilized.^49^^,^^50^^,^^51^ Coral tissues are both strong scatterers and absorbers of light, and variations in their optical properties significantly influence coral photophysiology.^28^^,^^51^^,^^52^^,^^53^ To fully understand these interactions, future work will require realistic 3D reconstructions of coral tissue architecture combined with accurate measurements of tissue-specific optical properties. Such models will help advance our understanding of coral photophysiology and improve predictions of how corals respond to changing light environments.

### Conclusions

Coral responses to light operate across multiple spatial and temporal scales, involving changes in coral morphology as well as modulations in both skeletal and tissue optical properties. It is well established that, at the colony scale, morphological traits (e.g., growth form) are excellent predictors of coral function within reef ecosystems,^54^ particularly in shaping coral communities across diverse light environments.^32^ Our study provides new insights into the relationships between coral micromorphological traits and light distribution, emphasizing the critical role of coral skeletal morphology in optimizing light capture at the microscale. The intricate architecture of corallite and coenosteum, combined with the scattering properties of the coral skeleton, facilitate the creation of microhabitats that balance light capture and shading, which could support photosynthesis by the endosymbiotic algae while minimizing photodamage. We found that coenosteum grooves effectively enhance light in their reflective convex spaces, enabling repeated light interactions to potentially enhance absorption by algal symbionts. While our findings support the role of skeletal optical properties in depth-specific light optimization, changes in skeletal microstructure could potentially result from physiological trade-offs between growth rate and calcification under different environmental pressures, with optical properties being secondary consequences rather than adaptive traits. Our study focused on *T. reniformis*, and variations in optical adaptations may exist among different coral species and across depth gradients is central to predicting and managing light-related stress phenomena, such as coral bleaching. Coral optics may thus provide insight into species-specific susceptibility to bleaching and other environmental stressors. As climate change continues to alter reef environments, this knowledge is increasingly vital for efforts to protect and restore coral reef ecosystems.

### Limitations of the study

This study focused on the skeletal optical properties of *T*. *reniformis*, excluding the influence of overlying tissues, which also modulate light propagation and absorption by symbiotic algae.^13^^,^^48^ Future work should incorporate tissue-specific optical properties and realistic 3D reconstructions to better capture *in vivo* light micro-environments.^27^ Additionally, while we identified two optically distinct skeletal layers, the study did not include direct nanoscale structural or compositional analyses (e.g., aragonite crystal orientation or density) that could clarify the physical basis of these differences. Finally, although our findings in *T. reniformis* are compelling, optical and structural adaptations may differ substantially across coral species and depth zones, limiting the broader generalizability of our conclusions.

## Resource availability

### Lead contact

Further information and requests should be directed to and will be fulfilled by the lead contact, Daniel Wangpraseurt (dwangpraseurt@ucsd.edu).

### Materials availability

This study did not generate new materials.

### Data and code availability

• Data: Raw data are available in the Dryad Digital Repository link: https://doi.org/10.5061/dryad.41ns1rnr9.
• Code: Original codes generated are available in the Dryad Digital Repository link: https://doi.org/10.5061/dryad.41ns1rnr9.
• Other: Any additional information required to reanalyze the data reported in this paper is available from the lead contact upon request.

## Acknowledgments

We thank the staff at the IUI for their assistance during the fieldwork. We also thank the National Center for Microscopy and Imaging Research (NCMIR) at the University of California, San Diego, for their technical assistance with μCT. We further acknowledge the support of research assistance from the Coral Reef Ecophysiology and Engineering lab. This work was supported by the Joint United States National Science Foundation (NSF) and United States – Israel Binational Science Foundation (NSF-BSF) grant no. 2021647 to Y.L., no. 2149925 to M.T. and D.W., and in part by funds from the Company of Biologists Fellowship (no. 2202660; sponsored by the Journal of Experimental Biology) to N.K.

## Author contributions

N.K. and D.W. conceptualized the study and designed the methodology. N.K. obtained the coral samples. N.K. and C.T.G.-M performed optical measurements. N.K. and S.L.J. wrote the OCT calibration and optical extraction codes. N.K. performed 3D light simulations. Y.L., M.T., and D.W. supervised the study. N.K. generated the figures, performed data analysis, and led the writing of the manuscript with contribution and final approval for publication from all authors.

## Declaration of interests

The authors declare no competing interests.

## STAR★Methods

### Key resources table

**Table:** 

REAGENT or RESOURCE	SOURCE	IDENTIFIER
**Biological samples**		

*Turbinaria reniformis*	Gulf of Aqaba/Eilat reefs, Northern Red Sea (29°30′05.9″*N* 34°55′01.7″E)	Nature and Park Authority, Israel, permit no.: 2020/42649

**Deposited data**		

Dryad Digital Repository	https://doi.org/10.5061/dryad.41ns1rnr9	https://doi.org/10.5061/dryad.41ns1rnr9

**Software and algorithms**		

R	R Core Team	Version 4.4.0
Dragonfly	Comet Technologies Canada Inc	Version 2024.1
MATLAB	MathWorks, Inc.	Version R2024a

**Other**		

Micro-Computed Tomography (μCT)	Carl Zeiss Microscopy GmbH	Xradia VersaXRM-510
Optical Coherence Tomography (OCT)	Thorlabs, Inc.	GAN311 Ganymede
DiagPolyTM Plain Polystyrene Nanoparticles, 100 nm	CD bioparticles	DNG-P259
Spectrometer	Ocean Insight	Flame
Reflectance probe	Ocean Insight	0.23 diameter

### Experimental model and study participant details

The depth generalist coral *Turbinaria reniformis*, known for its distinctive "leaf-like" morphology, was selected for this study because its abundance across depth gradients in the Gulf of Eilat/Aqaba, Red Sea.^12^^,^^55^ A total of ten corals were collected via SCUBA and technical diving, with five samples from shallow (5–10 m) and five from mesophotic (40–50 m) depths. To remove tissue, the colonies were bleached in a 6% sodium hypochlorite solution for 24 h, thoroughly rinsed with deionized water to remove residual organic matter, and air-dried at room temperature for an additional 24 h.

### Method details

#### OCT imaging

The 3D structural and optical information of coral corallites (Table 1) was extracted from tomographic measurements of the relative backscatter intensity using an OCT system (GAN311 Ganymede OCT Base Unit, Thorlabs, Inc.) equipped with a Thorlabs LSM03 objective scan lens. OCT cross-sectional images (or B-scans; ‘*z*’ vs. ‘*x*’ view) were acquired at a fixed pixel size of 1024 × 681, whereas the actual field of view was variable in *x* but fixed at *z* = 2 mm (i.e., resolution of 2.94 μm per pixel). Scans were performed in air to maximize signal contrast, and the refractive index was adjusted accordingly in the calculations. The sampling rate was set at 40 kHz. The effective Numerical Aperture (NA) is 0.075, and the OCT’s coherence length is 5.5 μm in air and 4.1 μm in water.

To calibrate the OCT system, we used known reference interfaces (glass/air, glass/water, and glass/oil) to determine the specular reflectance (*R*_*sp*_) values at these boundaries, following the methodology described by Wangpraseurt et al.^27^ *R*_*sp*_ was calibrated via Fresnel’s equations for normal incidence of light using the refractive index of air (n = 1), water (n = 1.33), oil (n = 1.46) and glass (n = 1.52). Additionally, we created a calibration curve using five concentrations of plain polystyrene nanoparticles (100 nm diameter; CD Bioparticles, USA; n = 1.59), each dispersed in deionized water at concentrations ranging from 2.84×10^12^ to 4.55×10^13^ spheres per mL (Figure S1). Theoretical optical properties were derived from Mie theory scattering calculations.^56^

To ensure accurate representation of variations in signal intensity arising from the focusing characteristics of the OCT system, the surface of the skeleton was placed at the focal point of the OCT system, allowing optimal measurements within a ±100 μm superficial region of the skeleton, thereby mitigating the effects of depth-dependent sensitivity fall-off. Finally, we performed a background scan without a sample to isolate and remove noise artifacts. Using these calibrations, we generated a function for measuring the reflected coherent light (*R*) of a given OCT signal (A-scan) measurement within the skeleton, which was recorded in log-encoded units of OCT(z) [counts] (Equation 1; Figure S1; a is the intercept and b is the slope):

(Equation 1) OCT(z)=a+b×ln(R(z))

#### Extraction of inherent optical properties

Data collection included at least 1500 OCT signal scans per skeletal sample. Specific regions of interest were selected to generate the signal profiles as a function of depth (*z*; in millimeters), from core skeletal features of the corallite and coenosteum, including the columella, septa, coenosteum grooves, and coenosteum spines. The signal profiles OCT(z) were calibrated to yield reflectance profiles R(z), which were analyzed by least-squares regression to yield a y-intercept at the skeleton surface (y-int) and a round-trip two-way attenuation (slope [1/mm]). Analysis converted y-int, slope into the local reflectivity (*ρ*) and the total attenuation coefficient (*μ* = -slope/2 [1/mm]) (Equation 2; Figures 1A–1D). To minimize artifacts from the air-skeleton interface, the first five data points following the maximum reflectance peak were excluded from the analysis, as these points may not accurately characterize subsurface properties. The slope fit was then extrapolated to the position of maximum reflectance to determine *ρ and μ*.

(Equation 2) $$R\left(z\right)={\rho e}^{-2\mu z}$$

To capture regional differences in attenuation and determine transition points, each signal profile was segmented into depth intervals using a piecewise linear fitting, and a linear fit was applied to each segment individually. The extraction of the optical scattering properties – specifically, the scattering coefficient (*μ*_*s*_; mm^−1^) and the anisotropy of scatter (*g*; dimensionless), were derived from the observed one-way attenuation (*μ*; mm^−1^) and reflectivity (*ρ*) following the relations:

(Equation 3) $$\mu =\left(a\left(g\right)\mu_{s}+\mu_{a}\right)G\left(NA\right)$$

(Equation 4) $$\rho =\mu_{s}L_{cg}b\left(g,NA\right)$$

For the calculation of *μ* (Equation 3), the factor a(*g*) (Figure S2A) decreases from 1 to 0 as g increases from 0 to 1, thereby reducing the contribution of *μ*_*s*_ to attenuation. This reduction limits the ability of *μ*_*s*_ to prevent incident light from reaching the OCT focal region within the skeleton or from being collected following backscattering at the OCT coherence gate as scattering becomes increasingly forward-directed (i.e., high *g*). Additionally, the absorption coefficient *μ*_*a*_ (mm^−1^), attributed to water, may contribute to attenuation. The factor G(NA) is approximately 1.0 for the OCT system, given its low numerical aperture (NA = 0.075). For the calculation of *ρ* (Equation 4), *L*_*cg*_ (mm) denotes the length of the coherence gate, and the term *μ*_*s*_*L*_*cg*_ represents the fraction of incident photons scattered within this region. The factor b(*g*, NA) accounts for the proportion of this scattered light that undergoes backscattering into the collection lens of the OCT system. As shown in Figure S2B (*g*, NA) decreases from 1.04 × 10^−4^ to 0 as *g* increases from 0 to 1, as forward-directed scattering does not return to the OCT system. The parameters *μ*_*s*_ and *g* are influenced by the concentration and size distribution of scatterers (nano- and sub-micron scale) within the skeleton. The absorption coefficient was set to 0.01 mm^−1^, assuming negligible light transmission through the coral skeleton at 930 nm for skeletons with a water content of 58%.^27^

Additionally, the surface complexity of the selected 3D regions (4 × 4 mm planar area) was quantified by calculating the rugosity index, obtained by dividing the actual 3D surface area by its corresponding planar area (Figure S3). All OCT image analyses were performed using MATLAB (v. R2024a; The MathWorks, Inc.), with scripts provided in the Supplementary Materials.

#### μCT imaging

High-resolution micro-computed tomography (μCT) was used on the bare coral skeletons as a non-destructive 3D digital visualization, offering the detection of intricate external and internal morphological details at a microstructural level. μCT leverages sequential 2D radiographic projections to reconstruct volumetric datasets, enabling quantitative analysis of fine-scale skeletal features without physical sectioning. The scans were conducted with a Zeiss Xradia VersaXRM-510 (Carl Zeiss Microscopy GmbH) 3D X-ray Microscope at the National Center for Microscopy and Imaging Research, University of California San Diego. The coral skeletons were scanned in a 360° rotation at an isotropic voxel size of 12 μm, voltage of 112 kV, amperage of 110 μA, and exposure time of 2s. Scans were saved in a TIFF image format for 3D volume rendering and quantitative analysis using Dragonfly software (© 2024 Object Research System (ORS) Inc.). Finally, we facilitated detailed surface renderings and internal volume quantification necessary for light-simulation 3D mesh generation (STL format).

#### Preprocessing, preparation, and execution of Monte Carlo simulations

All 3D mesh models were cropped to a 100 mm^2^ planar area, analyzed for defects such as unconnected vertices, and repaired as necessary. The cropped STL file was uploaded, and its dimensions were calculated to determine the pixel dimensions required for accurate voxelization (i.e., conversion into voxel format). Previous work utilizing MC modeling has been applied in either 2D or 3D voxel space, featuring basic computer-designed geometries.^8^^,^^19^ Next, we voxelized a 3D mesh from the STL file, creating a grid of small cubic volumes that represented the coral structure. The resolution was specified as 300 pixels per cm. After generating the coral model in voxel format, we divided the voxelized model into two layers: 1) a 100-micron superficial layer and 2) the deeper volumetric (subsurface) skeletal layer, assuming more homogeneous scattering due to reduced surface irregularities. Each voxel within the skeletal layers was then assigned specific optical properties obtained from the OCT scans. In addition, we added a water layer of 100 pixels above the coral surface to examine how the amount of light is reflected from the skeleton to the water.

Each simulation was run using 75×10^6^ virtual photons. The photon transport simulation involved launching photons with a wavelength of 930 nm and initiating their position and trajectory normal to the coral surface. For our MC simulations, we utilized the ‘mcxyz.c’ program.^29^ The MC method was used to predict the fluence rate distribution, defined as the total photon flux passing through a unit area from all directions per unit time, by simulating the light as random individual photons from a given light source. Although corals can experience varying light angles throughout the day under natural conditions, for simplicity, we set the angle of light as parallel rays perpendicular to the coral surface. Each photon has a chance to be absorbed, scattered, or passed through the material at a rate that is dependent on the local optical properties of the material. Upon completion, the simulation accumulated the status of all photon energies and produced a distribution of absorbed energy in each voxel which was then converted into the light fluence rate distribution. The fluence rate values were normalized per pixels (i.e., the sample’s skeletal size). For each simulation and layer, a histogram of the fluence rate was generated and the mean, median, variance, 95^th^ percentile, range, skewness and kurtosis were calculated.

#### Reflectance measurements

Diffuse spectral reflectance was measured to validate the simulation results.^9^^,^^57^ The samples were placed in a black acrylic chamber and illuminated with homogeneous diffuse light from a semi-sphere coated with barium oxide (BaO) and a LED lamp (CRI-MAX TM PAR 30, Yuji Lighting). Reflectance was measured using a flat-cut fiber-optic reflectance probe (diameter = 0.23 cm, Ocean Insight) connected to a miniature spectrometer (Flame, Ocean Insight; *n* = 5 scans per measurement, boxcar width = 2 nm, resolution = 0.2 nm). The probe was positioned 5 mm from the skeletal surface at a 45° angle relative to the surface. Three random surface regions per sample were selected for the measurements, and the experimental results were normalized against a 99% diffuse reflectance standard (Spectralon, Labsphere).

### Quantification and statistical analysis

Statistical analyses were conducted using the R software.^58^ Given that OCT measurements are susceptible to signal fluctuations, which can introduce variability, median values were used instead of means to reject outlier data and yield a robust representation of the data. Differences between depth and skeletal feature (fixed effects) were tested using a mixed-effects permutational analysis (MEPA; 999 permutations), and models included the coral’s colony ID as a random effect. These analyses were performed using the {lme4}^59^ and {predictmeans}^60^ packages. When significant differences were found, the standardized effect sizes and 95% confidence intervals (CI; 5000 bootstrap samplings) of the pairwise comparisons were estimated by calculating Hedges' g (*Hg*) using the package {dabestr}.^61^ CIs that did not overlap with zero were considered significant and were noted with an asterisk on relevant figures.
