# Supplementary material for: Depth-dependent microskeletal features modify light harvesting in Turbinaria reniformis corals
Source: iScience. 2025 Jul 17;28(8):113137. doi: 10.1016/j.isci.2025.113137 (PMC12329508; doi:10.1016/j.isci.2025.113137)
Supplement: Document S1. Figures S1–S6 and Table S1 [file mmc1.pdf]

iScience, Volume *28*

Supplemental information

Depth-dependent microskeletal features modify light harvesting in *Turbinaria reniformis* corals

Netanel Kramer, Claudia Tatiana Galindo-Martínez, Steven L. Jacques, Martin Tresguerres, Yossi Loya, and Daniel Wangpraseurt

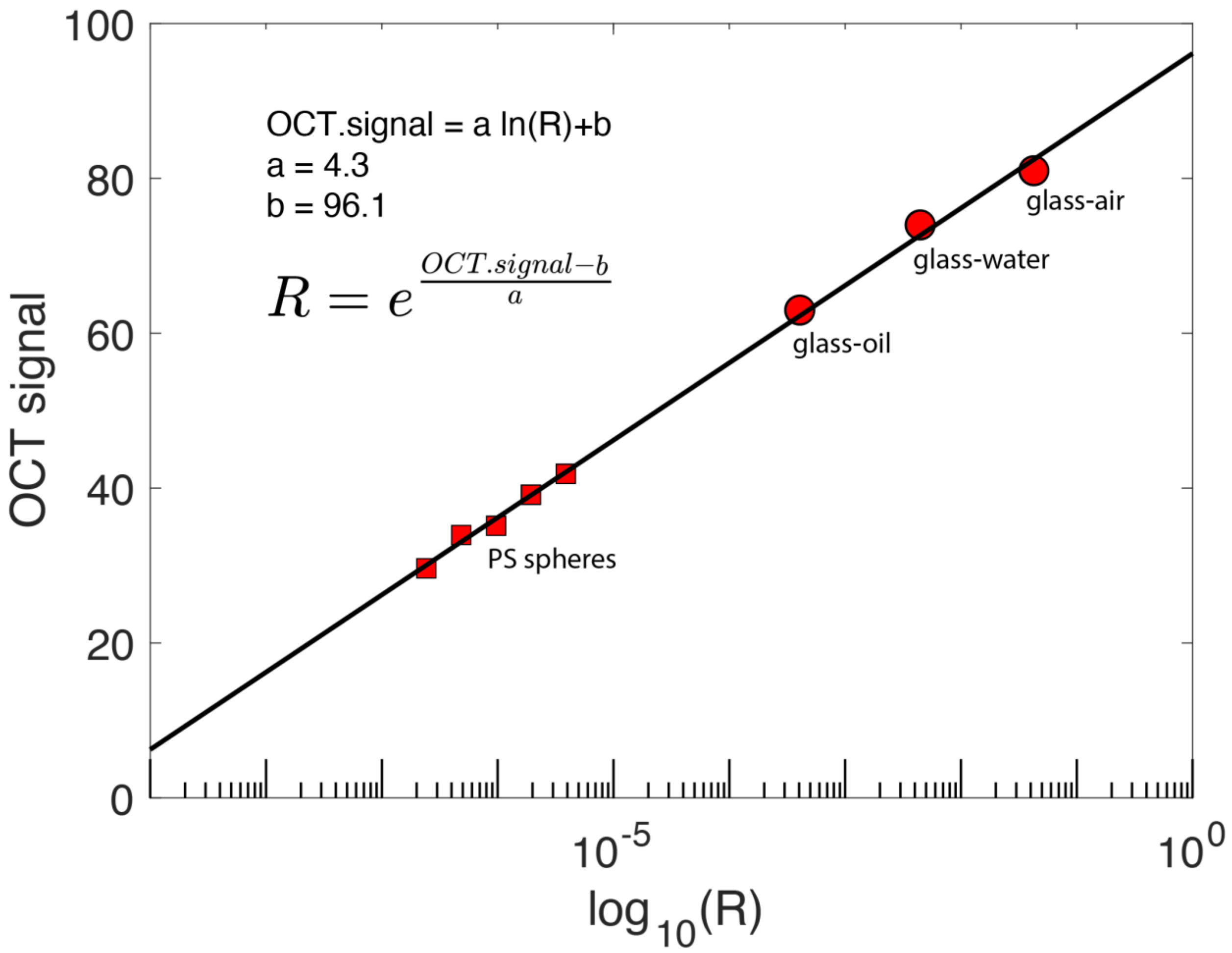

Figure S1. The calibration of the OCT system. The expected reflectance is plotted as *R* on the x-axis. The measured y-intercept of the polystyrene microspheres (square points) and the measured OCT of the air/glass, water/glass, and oil/glass interfaces are plotted on the y-axis. The expected reflectances of the air/glass, water/glass, oil/glass interfaces. The black line through the data serves as a calibration of the OCT system.

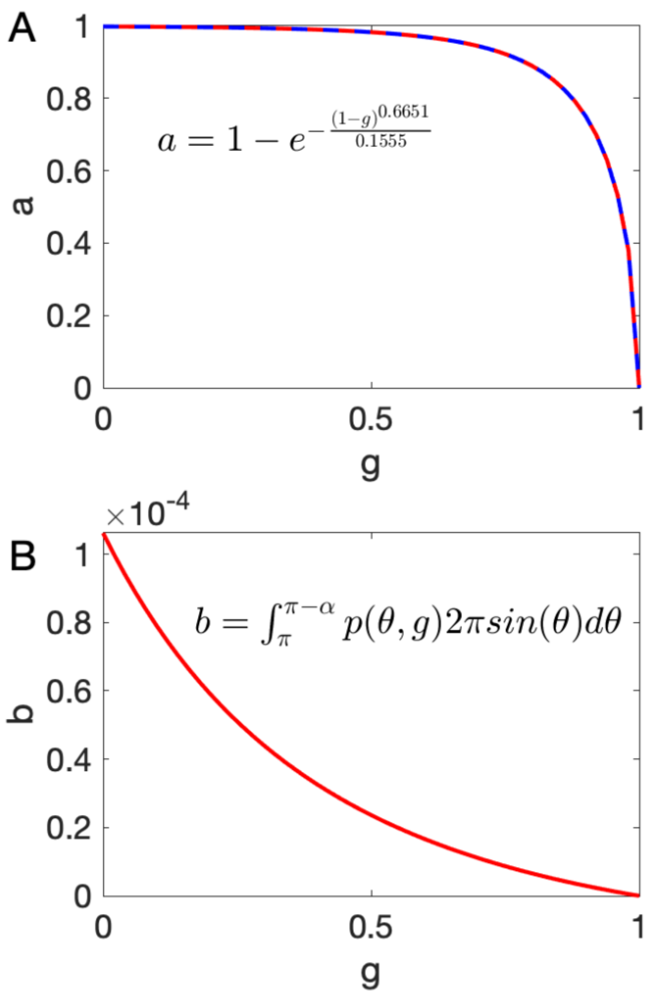

Figure S2. Monte Carlo simulation results for scattering functions in OCT imaging. (a) The function *a(g)* and **(b)** the function *b(g)*, based on Monte Carlo simulations. The scattering function *p(θ, g)* [1/sr] uses the Henyey-Greenstein function, where θ is the deflection angle of scatter. The half-angle of collection by the OCT system equals 3.88 radians (3.87°): α = arcsin(NA/*n*), NA = 0.075, n = refractive index mismatch = 0.057 (using n_{air} = 1 and $n_{skeleton}$ = 1.65)[1].

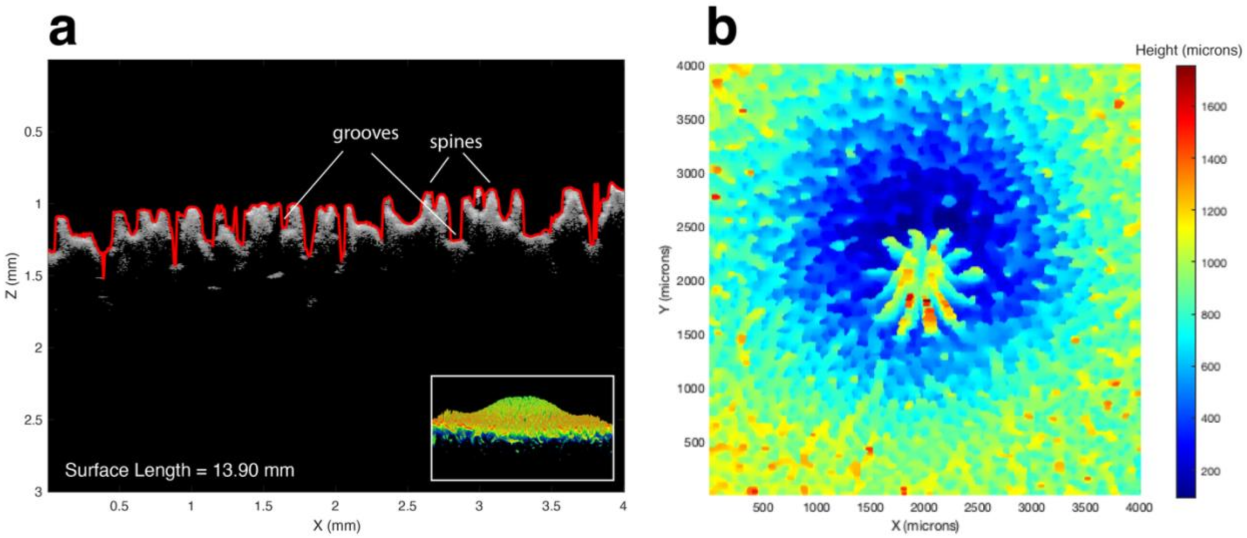

Figure S3. Example of visualization of surface topography analysis. (a) Cross-sectional OCT scan showing the detected and processed surface boundary (*red*) extracted from TIFF image stacks. Inlet image is the 3D OCT scan from which the slice was taken. **(b)** 3D scattered point cloud visualization of the complete surface topography, where color intensity represents the Z-axis height in microns. Total surface area was calculated by performing Delaunay triangulation on the point cloud and summing the areas of all resulting triangles.

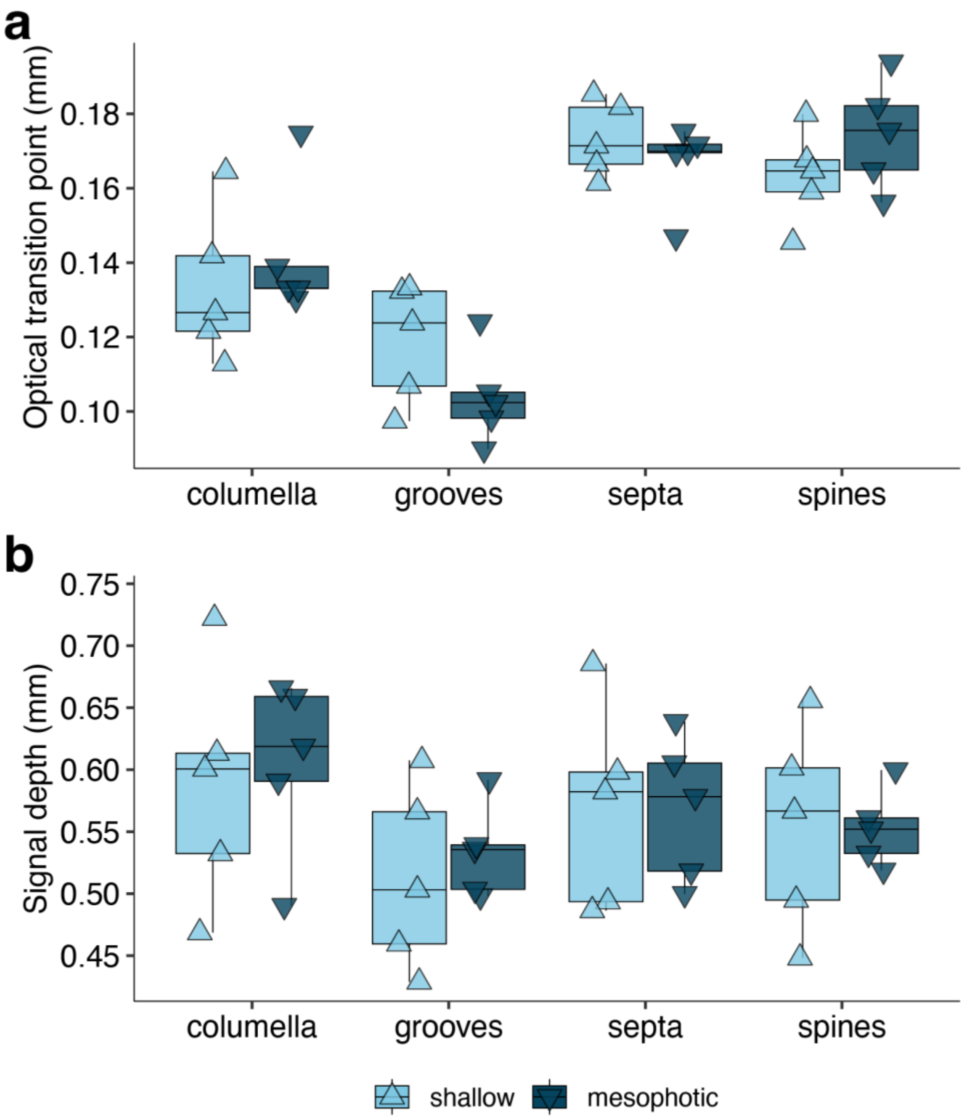

Figure S4. Optical characteristics and penetration depth analysis of shallow and mesophotic *T. reniformis*. (a) A parameter denoting the point of significant alteration in the optical attenuation coefficient (μ), defined as "optical transition point" (in mm) between shallow (*light blue*; triangle point up) and mesophotic (*dark blue*; triangle point down) *T. reniformis*, and among its skeletal features. **(b)** The maximum penetration depth at which the backscattered signal intensity intersects with the OCT system's noise floor. Each triangle represents the median of a sample. Box plots horizontal lines depict the median, box height depicts the interquartile range, whiskers depict ±1.5× interquartile range.

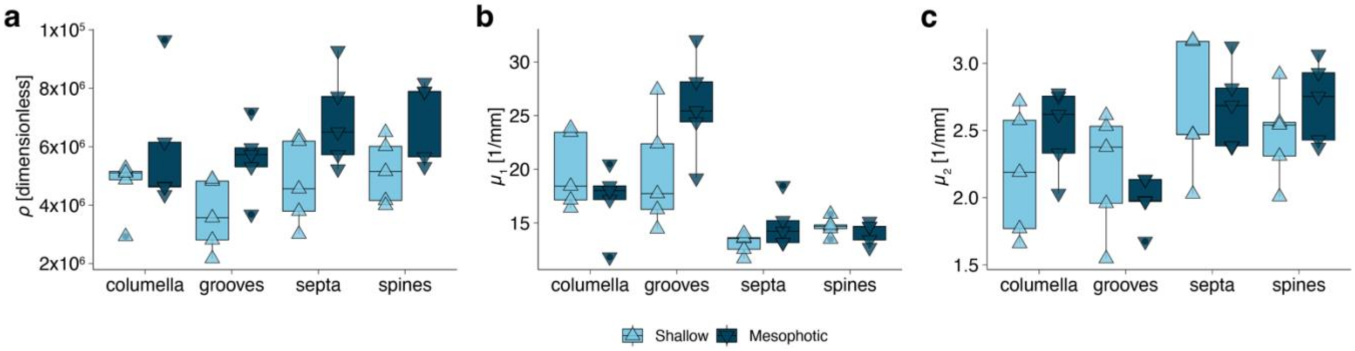

Figure S5. OCT-derived optical parameters of skeletal components in shallow and mesophotic T. reniformis. The extracted **(a)** ρ (local reflectivity), **(b)** μ_1 (total attenuation) for the superficial skeletal layer and **(c)** μ_2 for the volumetric skeletal layer from the OCT signal attenuation of morphological skeletal components between shallow (*light blue*; triangle point up) and mesophotic (*dark blue*; triangle point down) *T. reniformis*. Each triangle represent the median of a sample. Box plots horizontal lines depict the median, box height depicts the interquartile range, whiskers depict ±1.5× interquartile range.

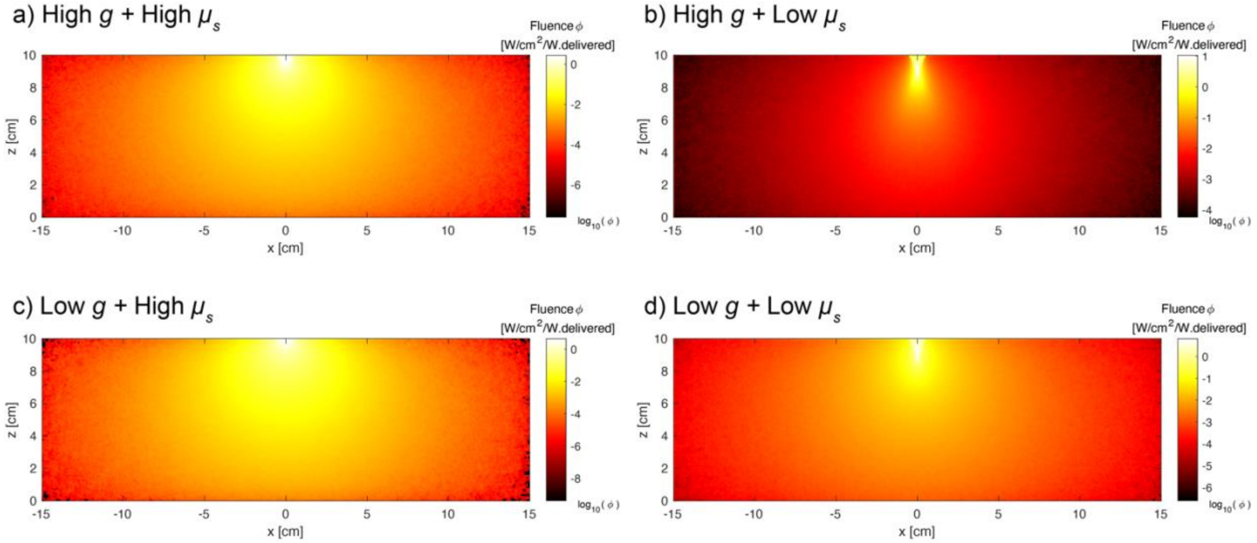

Figure S6. **Spatial distribution of the relative fluence rate ($\log_{10}(\phi)$) on a 2D cross-section as a function of optical properties**. **(a)** high g + high μ_s; **(b)** high g + low μ_s; **(c)** low g + high μ_s; and **(d)** low g + low μ_s. The color bar indicates the logarithm of fluence rate in units of W/cm^2 per unit power delivered.

Table S1. Statistical summary table of log-scaled fluence rate distributions across coral layers (volumetric and superficial) and water column in shallow and mesophotic 3D *Turbinaria reniformis* samples.

Layer	Depth	Sample	Mean	Median	Variance	p95	Range	Skewness	Kurtosis
Volumetric layer	Shallow	1	-0.37	-0.22	0.44	0.67	8.70	-1.13	4.51
Superficial layer	Shallow	1	0.37	0.41	0.05	0.22	2.81	-2.18	11.53
Water	Shallow	1	-0.43	0.10	0.94	0.97	8.50	-1.09	2.58
Volumetric layer	Shallow	2	-0.09	0.01	0.18	0.43	4.01	-0.89	3.30
Superficial layer	Shallow	2	0.34	0.40	0.05	0.23	2.68	-2.20	10.40
Water	Shallow	2	-0.09	0.11	0.23	0.48	6.67	-1.50	4.36
Volumetric layer	Shallow	3	-0.34	-0.27	0.32	0.56	8.54	-0.84	3.85
Superficial layer	Shallow	3	0.35	0.43	0.07	0.26	3.95	-2.31	9.37
Water	Shallow	3	-0.26	0.10	0.46	0.68	7.70	-1.28	3.58
Volumetric layer	Shallow	4	-0.11	0.00	0.23	0.48	6.81	-1.19	4.84
Superficial layer	Shallow	4	0.40	0.43	0.03	0.17	2.57	-2.16	11.62
Water	Shallow	4	-0.11	0.15	0.30	0.55	7.31	-1.25	3.56
Volumetric layer	Shallow	5	-0.34	-0.28	0.25	0.50	6.35	-0.67	3.32
Superficial layer	Shallow	5	0.39	0.42	0.03	0.16	2.62	-1.44	7.00
Water	Shallow	5	-0.02	0.16	0.28	0.53	7.41	-2.19	7.79
Volumetric layer	Mesophotic	1	-0.17	-0.12	0.15	0.39	2.70	-0.51	2.53
Superficial layer	Mesophotic	1	0.36	0.37	0.01	0.12	1.92	-1.66	11.53
Water	Mesophotic	1	-0.08	0.11	0.21	0.46	4.59	-1.51	3.96
Volumetric layer	Mesophotic	2	-0.23	-0.19	0.20	0.44	6.38	-0.56	3.07
Superficial layer	Mesophotic	2	0.36	0.41	0.05	0.22	3.45	-2.60	14.45
Water	Mesophotic	2	-0.02	0.14	0.22	0.47	6.88	-2.02	6.91
Volumetric layer	Mesophotic	3	-0.27	-0.21	0.16	0.40	5.33	-0.85	4.24
Superficial layer	Mesophotic	3	0.24	0.27	0.05	0.22	2.80	-0.88	4.47
Water	Mesophotic	3	-0.04	0.11	0.19	0.44	6.56	-2.09	6.95
Volumetric layer	Mesophotic	4	-0.25	-0.16	0.32	0.57	6.92	-0.73	3.16
Superficial layer	Mesophotic	4	0.46	0.54	0.06	0.24	4.35	-2.88	15.31
Water	Mesophotic	4	-0.27	0.18	0.91	0.95	8.35	-1.47	3.66
Volumetric layer	Mesophotic	5	-0.17	-0.10	0.28	0.53	7.26	-0.77	3.41
Superficial layer	Mesophotic	5	0.48	0.56	0.06	0.25	3.55	-3.13	15.01
Water	Mesophotic	5	-0.02	0.21	0.45	0.67	7.23	-2.14	6.44

References

1. Farfan, G.A., Apprill, A., Cohen, A. et al. Crystallographic and chemical signatures in coral skeletal aragonite. Coral Reefs 41, 19–34 (2022). https://doi.org/10.1007/s00338-021-02198-4
